# Auxiliary Value of [^18^F]F-Fluorocholine PET/CT in Evaluating Post-Stereotactic Radiosurgery Recurrence of Lung Cancer Brain Metastases: A Comparative Analysis with Contrast-Enhanced MRI

**DOI:** 10.3390/cancers17152591

**Published:** 2025-08-07

**Authors:** Yafei Zhang, Mimi Xu, Shuye Yang, Lili Lin, Huatao Wang, Kui Zhao, Hong Yang, Xinhui Su

**Affiliations:** 1 Department of Nuclear Medicine, The First Affiliated Hospital, Zhejiang University School of Medicine, Hangzhou 310003, China; zhangyafei@zju.edu.cn (Y.Z.); 1520008@zju.edu.cn (M.X.); yangshuye@zju.edu.cn (S.Y.); 1511005@zju.edu.cn (L.L.); 1710005@zju.edu.cn (H.W.); zhaokui0905@zju.edu.cn (K.Z.); 2 Department of Radiology, The First Affiliated Hospital, Zhejiang University School of Medicine, Hangzhou 310003, China

**Keywords:** brain metastasis from lung cancer, PET/CT, [^18^F]F-fluorocholine, contrast-enhanced MRI, stereotactic radiosurgery

## Abstract

Background/Objectives: This study aims to evaluate the additional value of [^18^F]F-fluorocholine ([^18^F]F-FCH) PET/CT over contrast-enhanced magnetic resonance imaging (CE-MRI) in detecting the recurrence of brain metastases (BMs) after stereotactic radiosurgery (SRS) in patients with lung cancer brain metastases (LCBMs). Methods: Thirty-one patients with suspected recurrence of BM in LCBM after SRS were enrolled in this retrospective study. They underwent both [^18^F]F-FCH PET/CT and CE-MRI within 2 weeks. The tumor imaging parameters and clinical features were analyzed. The results of histopathology or radiographic follow-up served as the reference standard for the final diagnosis. Results: In these 31 patients, there were 54 lesions, of which 27 lesions were proven to be BM recurrence, while 27 lesions were non-recurrence. [^18^F]F-FCH PET/CT showed high radiotracer uptake in recurrent lesions of BM and identified 24 positive lesions (88.89% of sensitivity), while CE-MRI indicated 23 positive lesions (85.19% of sensitivity). [^18^F]F-FCH PET/CT indicated higher specificity (81.48%) and accuracy (85.19%) in detecting recurrence of BM than CE-MRI (40.74% and 62.96%, both *p* < 0.05), particularly in frontal lobes and cerebella. For lesion sizes, the accuracy of [^18^F]F-FCH PET/CT in detecting recurrent lesions was higher than that of CE-MRI for lesions over 1.0 cm but below 2.0 cm (*p* = 0.016). The detective performance of [^18^F]F-FCH PET/CT combined with CE-MRI was higher than [^18^F]F-FCH PET/CT or CE-MRI alone (all *p* < 0.05). Interestingly, TLC (≥4.11) was significantly correlated with poor intracranial PFS (iPFS), meaning it was a significant prognostic factor for iPFS. Conclusions: This study identified that compared with CE-MRI, [^18^F]F-FCH PET/CT demonstrated higher specificity and accuracy in diagnosing recurrence of BM in LCBM after SRS. Combining [^18^F]F-FCH PET/CT with CE-MRI has the potential to improve diagnostic performance for recurrence of BM and management of patient treatment. TLC was an independent risk factor for iPFS.

## Simple Summary

Conventional imaging examinations have limitations in diagnosing brain tumor recurrence. [^18^F]F-fluorocholine ([^18^F]F-FCH PET/CT) showed lower uptake in normal brain parenchyma and higher tumor-to-brain contrast. There are few data on the use of [^18^F]F-FCH PET/CT for detecting brain tumor recurrence. This study evaluated the effectiveness of [^18^F] F-FCH PET/CT versus contrast-enhanced MRI (CE-MRI) in detecting brain metastasis recurrence after stereotactic radiosurgery (SRS) in 31 lung cancer patients. Among 54 lesions analyzed, [^18^F]F-FCH PET/CT demonstrated higher specificity and accuracy than CE-MRI. For lesions sized 1.0–2.0 cm, PET/CT outperformed MRI significantly. Combining both methods improved diagnostic performance compared to either alone. Additionally, total lesion choline uptake (TLC) was linked to shorter intracranial progression-free survival (iPFS), identifying it as a prognostic marker. [^18^F]F-FCH PET/CT is a promising imaging modality for detecting the recurrence of brain metastases in lung cancer patients after stereotactic radiosurgery. Combined [^18^F]F-FCH PET/CT and CE-MRI enhance diagnostic accuracy and patient management in brain metastasis recurrence after SRS.

## 1. Introduction

Brain metastasis (BM) occurs in approximately 20–30% of cancer patients [1,2,3] and has become a major cause of patient morbidity and mortality. It has been reported that lung cancer patients have high BM rates, accounting for 67–80% of such cases [3,4]. There are three main treatment methods for BM: radiation therapy, surgery, and anti-tumor drug treatment. The latter option has rather limited efficacy to suppress BM progression since anti-tumor drugs are restricted from passing through the blood–brain barrier (BBB) [4,5]. As a form of radiation therapy, stereotactic radiosurgery (SRS) has been widely used to treat BM from various cancers due to its efficacy, short treatment time, and low neurocognitive toxicity [6,7]. Based on the American Society of Radiation Oncology (ASTRO) [8] and International Stereotactic Radiosurgery Society (ISRS) consensus guidelines [9], SRS has been recommended as a standard of care for patients with a good performance status and fewer than four BM lesions. Although SRS can reach more than 70% of the local control rate of BM [10], up to 50% of BM after SRS will develop either radionecrosis (RN) or tumor recurrence [11]. RN is among the most frequent adverse effects following SRS for brain metastases, occurring in 25% of patients [12,13]. Conventional imaging cannot reliably distinguish these entities, critically impacting treatment strategies [14,15]. Multimodal neuroimaging, including perfusion imaging and amino acid-based imaging, significantly improves differentiation between RN and tumor recurrence [16]. Therefore, early detection for the recurrence of BM after SRS is critical for prompt treatment and optimal control of the disease. 

Magnetic resonance image (MRI) is widely used to screen for BM due to the higher sensitivity than computed tomography (CT) in detecting the size, number, and distribution of brain lesions [16]. However, MRI in the diagnosis of BM is particularly challenging in cases of single metastasis and after surgery or radiation therapy, as it has shown a limitation in differentiating a brain tumor from a brain abscess or RN [17,18]. Therefore, a diagnostic tool with high specificity for diagnosing the recurrence of BM after SRS is urgently needed to meet the needs of clinicians. Some novel MRI sequences including perfusion-weighted imaging (PWI), MR spectroscopy (MRS), diffusion-weighted imaging (DWI), and MR chemical exchange saturation transfer (CEST) may help to compensate for these shortcomings [19,20]. Perfusion MRI can differentiate between RN and tumor recurrence by calculating cerebral blood flow in the lesion area, but paramagnetic artifacts and contrast agent extravasation may affect the accuracy of the results [21]. Some studies noted that DWI and the derived apparent diffusion coefficient (ADC) can differentiate between RN and tumor recurrence [22].

[^18^F]F-fluorodeoxyglucose ([^18^F]F-FDG) positron emission tomography/computed tomography (PET/CT) is now frequently being conducted as a major imaging option in cancer diagnosis, staging, and therapeutic monitoring. However, it has been shown that [^18^F]F-FDG PET/CT is relatively limited in terms of both the evaluation of the primary brain tumor and of possible recurrences due to low lesion contrast resulting from high normal physiological metabolism of the cerebral cortex. Hence, there is an urgent need to develop more sensitive novel PET probes to improve the diagnosis and staging of brain tumors based on their pathological characteristics. Amino acid tracers, including [^11^C]-methionine (MET), [^18^F]-fluoro-L-dihydroxyphenylalanine (F-DOPA), and [^18^F]fluoroethyl-L-tyrosine (FET), have yielded highly promising results, though each has its own strengths and limitations [23,24,25]. It is noteworthy that [^18^F]F-fluoroethyltyrosine ([^18^F]F-FET) currently serves as a commonly used neuro-oncological imaging agent. However, its application warrants further exploration due to the significant influence of blood–brain barrier disruption on tracer uptake [26].

Choline is a precursor used to biosynthesize phosphatidylcholine, an important structural component of the cell membrane. After being absorbed by the cells via specific transporters, choline is phosphorylated by the enzyme choline kinase, transformed into phosphatidylcholine, and converted into a component of the cell membrane [27,28]. Tumor cells with a high rate of proliferation present an increased need for choline due to a large number of cell membranes formed. Therefore, choline is generally overexpressed in tumor cells and has been used as a novel tumor imaging target [29,30]. In clinical practice, radionuclide ^11^C (carbon-11) or ^18^F (fluorine-18)-labeled choline, including [^11^C]-choline and [^18^F]-fluorocholine (or [^18^F]F-choline), has shown promising outcomes in the diagnosis of either various malignancies, such as gliomas, prostate cancer, and lung cancer, or benign conditions, including parathyroid adenomas [28,29,30]. Compared with ^11^C (half-life of 20 min), ^18^F possesses a longer half-life of 110 min and is more suitable for clinical application and transportation. It has been indicated that [^18^F]F-fluorocholine ([^18^F]F-FCH) PET/CT has a high diagnostic accuracy in brain tumors with a good specificity [31,32]. This retrospective study therefore aimed to evaluate the performance of [^18^F]F-FCH PET/CT in evaluating the recurrence of BM from lung cancer after SRS by comparing it with contrast-enhanced MRI (CE-MRI), and to determine the added value of [^18^F]F-FCH PET/CT in identifying brain metastatic lesions.

## 2. Materials and Methods

### 2.1. Patients

We retrospectively enrolled lung cancer brain metastasis patients referred to our department between March 2021 and December 2024, who had undergone SRS and were suspected of recurrence. All enrolled patients presented with oligometastatic intracranial disease (<4 lesions) and had undergone SRS at least 6 months prior to study enrollment. As documented, the administered peripheral dose ranged between 15 and 20 Gy, with treatment delivery standardized at the 45–50% isodose line. All enrolled patients underwent an [^18^F]-FCH PET/CT scan and conventional MRI, including fluid-attenuated inversion recovery (FLAIR), apparent ADC, and contrast-enhanced (CE) and non-CE T1-weighted sequences, within one month of initial characterization of suspected recurrent lesions. This retrospective study was approved by the institutional review board of the First Affiliated Hospital, Zhejiang University School of Medicine. Informed and written consent was obtained from all patients.

### 2.2. Brain CE-MRI Imaging and Analysis

CE-MRI imaging was performed on a 3.0 T MR unit (GE Discovery, General Electric Healthcare, Boston, MA, USA) equipped with an 8-channel head coil. All patients were scanned using the routine protocols including T1WI, T2WI, FLAIR, DWI, and ADC sequences. The FLAIR sequence was TR = 8800 ms, TE = 94 ms, TI = 2500 ms, FOV = 240 mm, matrix = 256 × 256, and slice thickness = 5.0 mm. The DWI sequence was as follows: TR = 3500 ms, TE = 98 ms, b = 0 and 1000 s/mm^2^, FOV = 240 mm, matrix = 160 × 160, and slice thickness = 5.0 mm. After a plain scan, enhanced MRI examination was performed after the injection of Gd-DTPA (0.15 mmol/kg) via the antecubital vein at a flow rate of 3.0 mL/s.

All MRI images were registered in the RSVSViewer (Radinfo Medical Science Co., Ltd., Hangzhou, China) and analyzed independently by two experienced radiology physicians blinded to the clinical data and pathological findings. Any difference in opinion between these two physicians was resolved by consensus. Nodules with abnormal enhancement on CE-MRI were considered positive lesions. By combining conventional MRI T1-weighted and CE-MRI images, an ROI with an area of 2–4 mm^2^ was selected on the brain metastatic lesion for measuring the ADC value, and each lesion was measured three times to calculate the average value.

### 2.3. [^18^F]F-FCH Synthesis

[^18^F]F-FCH was prepared as previously reported [33] and automatically synthesized in our center in a Good Manufacturing Practice (GMP) environment. Briefly, the choline reagent kit was purchased from Beijing PET Technology Co., Ltd. (Beijing, China). Fluorine-18 was produced on site using a Siemens Eclipse cyclotron (Siemens Medical Solutions, Knoxville, TN 37932, USA). The radiochemical synthesis of [^18^F]F-FCH was performed in an AllInOne^®^ synthesis module (Trasis, Ans, Belgium) following a procedure previously described [34]. Subsequently, the quality control of [^18^F]F-FCH was determined. The radiochemical purity of [^18^F]F-FCH was over 95%.

### 2.4. Brain PET/CT Imaging

A PET/CT scanner (Biograph version, Siemens, Erlangen, Germany) was used in this study. For all patients, PET scans were obtained 1 h after injection of [^18^F]F-FCH at a dose of 3.7 to 4.44 MBq (0.1–0.12 mCi)/kg. A PET scan was performed with 3 min per frame 3D acquisition. Low-dose brain CT (120 kV) was acquired to conduct attenuation correction, apply all necessary corrections to the reconstructed images, and provide anatomical information. All data were reconstructed using a Siemens workstation (syngo.via Client 4.1).

### 2.5. PET/CT Image Analysis

The results of [^18^F] F-FCH PET/CT scans were independently reviewed using volume viewer software on a Siemens workstation (Syngo.via Client 4.1) by two experienced nuclear medicine physicians who were blinded to the clinical data and pathological findings. Any discrepancies in interpretation between the two physicians were resolved through consensus. Any focal [^18^F]F-FCH uptake higher than the normal tissue background was considered a positive lesion. Based on 3D localization, the tumor region of interest (ROI) was manually delineated on axial images centered around areas of intense [^18^F]F-FCH uptake. Lesion [^18^F]F-FCH uptake was semi-quantified by maximum standardized uptake values (SUV_max_), tumor-to-background ratio (TBR), metabolic tumor volume (MTV), and total lesion choline uptake (TLC). MTV is defined as the volume of the ROI within a fixed threshold of 40% SUV_max_, while TLC is calculated as the product of MTV and the mean SUV value within this 40% fixed-threshold region.

### 2.6. Follow-Up and Outcome of Patients

The recurrence status was determined based on the diagnosis of tissue samples obtained from biopsies or tumor resections within 30 days after [^18^F]F-FCH PET acquisition. For patients who were unable to undergo biopsies for certain reasons, we conducted corresponding clinical and imaging follow-ups for at least one year. For these lesions, those showing obvious changes on CE-MRI during follow-up were identified accordingly. Diagnosis of recurrence requires integrating multimodal imaging, clinical assessment, and pathological verification. Pathological confirmation remains the gold standard for diagnosing tumor recurrence. Clinical evaluation necessitates a confirmation of new or worsening neuropsychiatric symptoms while excluding non-neoplastic factors such as cerebrovascular disease or infection. Regarding imaging assessment, progression is defined per the Response Assessment in Neuro-Oncology Brain Metastases (RANO-BM) criteria as either [35] (a) a greater than 20% increase in the sum of the products of perpendicular diameters for targets enhancing lesions on MRI, coupled with a minimum 5 mm absolute increase in lesion diameter during stable treatment, or (b) significant enlargement of T2 or FLAIR non-enhancing lesions after excluding radiation necrosis or infection. For patients with prior PET scans, recurrence is also indicated by either a ≥30% increase in the maximum target-to-background ratio (TBR_max_) or a ≥40% increase in metabolic tumor volume [36].

Moreover, further surgery or SRS was performed for recurrent lesions identified by follow-up, and regular laboratory and brain CE-MRI examinations were performed every 3–6 months after therapy for intracerebral lesions. The intracranial progression-free survival (iPFS) was measured from the time the intracranial recurrent lesion was managed to the next documented brain disease recurrence or progression. Patients without events were censored at the final clinical assessment in January 2023.

### 2.7. Statistical Analysis

Statistical analysis was performed using IBM SPSS version 29 (IBM, Armonk, NY, USA) and GraphPad Prism 9.0 (GraphPad Software, San Diego, CA, USA). The quantitative data were summarized using the mean and standard deviation (SD). The differences in diagnostic performance between [^18^F]F-FCH PET/CT and CE-MRI were analyzed using McNemar’s test and the Mann–Whitney U test. The iPFS times were calculated using Kaplan–Meier curves and compared with categorical variables using Cox analysis. A *p*-value of less than 0.05 was considered to show a statistically significant result. Receiver operator characteristic (ROC) curves were computed to find the optimal cut-off values.

## 3. Results

### 3.1. Patient Characteristics

The demographic and clinicopathological characteristics of 31 patients enrolled in our study are summarized in Table 1. Of them, there were 25 patients with BM from non-small-cell lung cancer (NSCLC) and 6 patients from small-cell lung cancer (SCLC). Moreover, 15 patients (48.39%) were asymptomatic, and 16 patients (51.61%) had clinical symptoms, including 8 patients with headache, 4 patients with nausea and vomiting, and 4 patients with seizures. Of all 54 lesions in 31 patients, histological confirmation was obtained for 12 lesions in 9 patients after [^18^F]F-FCH PET/CT, and the remaining were confirmed by imaging and follow-up. Of the 31 patients ultimately confirmed through histopathological examination or imaging follow-up, 23 exhibited recurrent lesions within their lesions, whereas no recurrent lesions were detected in 8 patients. Overall, 23 patients with 27 BM recurrent lesions underwent individualized treatment, including surgical treatment (*n* = 11) and gamma knife therapy (*n* = 12). During long-term follow-up, 10 of these 23 patients experienced intracranial recurrence or progression after the management of recurrent lesions.

### 3.2. Diagnostic Performance of [^18^F]F-FCH PET/CT and CE-MRI in Detecting Recurrence of BM in LCBM After SRS

**Figure 1:**
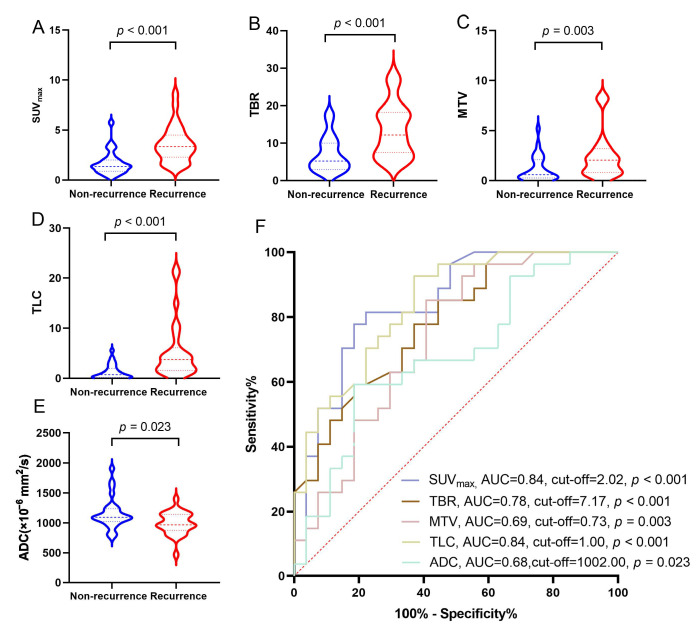
(**A**) Difference in SUV_max_ between recurrence and non-recurrence groups (*p* < 0.001); (**B**) difference in TBR between recurrence and non-recurrence groups (*p* < 0.001); (**C**) difference in MTV between recurrence and non-recurrence groups (*p* = 0.003); (**D**) difference in TLC between recurrence and non-recurrence groups (*p* < 0.001); (**E**) difference in ADC between recurrence and non-recurrence groups (*p* = 0.023); (**F**) ROC curves of [^18^F]F-FCH PET/CT and MRI parameters for predicting the efficacy of recurrence and non-recurrence.

**Figure 2:**
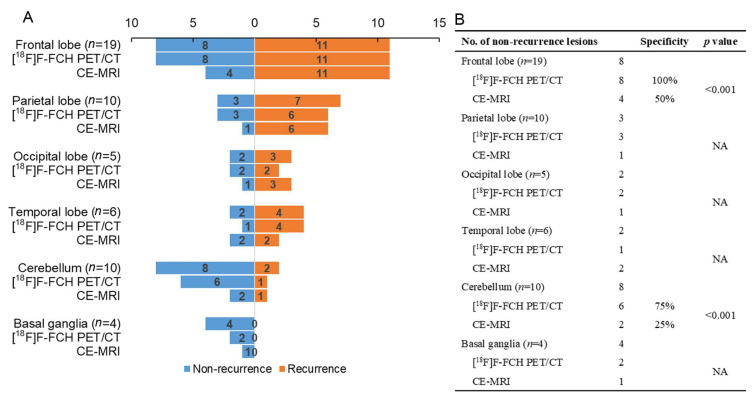
Comparison of diagnostic specificity of [^18^F]F-FCH PET/CT and CE-MRI in detecting lesions in different brain regions. (**A**) The numbers of patients with recurrent or non-recurrent lesions detected by [^18^F]F-FCH PET/CT and CE-MRI in different brain regions. (**B**) The diagnostic specificity of [^18^F]F-FCH PET/CT and CE-MRI in detecting lesions in different brain regions.

For the patient-based analysis, of these 31 patients, a total of 54 lesions were suspected recurrence of BM by [^18^F]F-FCH PET/CT or CE-MRI: 27 lesions (50.00%) were confirmed as recurrent BM, and 27 lesions (50.00%) were non-recurrent. The comparative results between [^18^F]F-FCH PET/CT and CE-MRI for detecting recurrence of BM in LCBM after SRS are shown in Table 2. [^18^F]F-FCH PET/CT showed high radiotracer uptake in the recurrent lesions of BM (Figure 1 and Figure 2) and found 24 positive lesions (88.89% sensitivity), and CE-MRI showed 23 positive lesions, mostly with low ADCs (85.19% sensitivity). [^18^F]F-PET/CT indicated higher specificity (81.48%) and accuracy (85.19%) in detecting recurrence of BM than CE-MRI (40.74% and 62.96%, *p* = 0.003, 0.008). The SUV_max_, TBR, MTV, and TLC in the recurrence groups were significantly higher than those in the non-recurrence groups (3.59 ± 0.35 vs. 1.69 ± 0.23, 13.44 ± 1.37 vs. 7.04 ± 0.91, 2.45 ± 0.44 vs. 1.16 ± 0.25, 5.37 ± 1.10 vs. 1.25 ± 0.28, respectively, all *p* < 0.05); meanwhile, ADCs in the recurrence groups were significantly lower than those in the non-recurrence groups (992.22 ± 35.92 vs. 1156.81 ± 50.12, *p* = 0.023) (Figure 1A–E). When the cut-off points of SUV_max_, TBR, MTV, TLC, and ADC were 2.02, 7.17, 0.73, 1.00, and 1002.00, respectively, [^18^F]F-FCH PET/CT had high sensitivity and specificity in differentiating recurrent from non-recurrent lesions (81.50% and 77.78%, 77.78% and 63.00%, 85.20% and 59.30%, 92.6% and 63.00%, 81.50% and 59.30%), respectively, and the areas under the curve (AUCs) were 0.84, 0.78, 0.69, 0.84, and 0.68 (Figure 1F).

### 3.3. Comparison of Diagnostic Performance of [^18^F]F-FCH PET/CT and CE-MRI in Detecting Lesion Distributions and Sizes of Recurrent BM

As for lesion-based evaluation, the results for [^18^F]F-FCH PET/CT and CE-MRI in the detection of lesions in different brain regions are shown in Figure 2. Most lesions were found in the frontal lobe (*n* = 19), cerebellum lobe (*n* = 10), and parietal lobe (*n* = 10). [^18^F]F-FCH PET/CT showed a higher specificity in detecting non-recurrent lesions than CE-MRI, such as lesions in the frontal lobe (100% [8/8] vs. 50% [4/8], *p* < 0.001) and cerebellum lobe (75% [6/8] vs. 25% [2/8], *p* < 0.001). However, they had similar sensitivity in detecting recurrent lesions. Moreover, the sensitivity, specificity, and accuracy of [^18^F]F-FCH PET/CT combined with CE-MRI in detecting recurrent lesions of BM were 96.26%, 85.19%, and 90.74%, respectively. The sensitivity was superior to that of [^18^F]F-FCH PET/CT alone (*p* = 0.004), the specificity was better than that of MRI (*p* < 0.001), and the accuracy was higher than that of [^18^F]F-FCH PET/CT alone (*p* < 0.001) or CE-MRI alone (*p* < 0.001).

In addition, [^18^F]F-FCH PET/CT showed five false-positive lesions attributed to RN: two in the temporal lobe, two in the cerebellum, and one in the basal ganglia. Three false-negative lesions were observed due to a partial volume effect, involving the parietal lobe, occipital lobe, and cerebellum. CE-MRI demonstrated 16 false-positive lesions attributed to RN or scar tissue: 2 in the parietal lobe, 4 in the frontal lobe, 1 in the occipital lobe, 3 in the basal ganglia, and 6 in the cerebellum. Four false-negative lesions were identified on CE-MRI: one in the parietal lobe, two in the temporal lobe, and one in the cerebellum.

**Figure 3:**
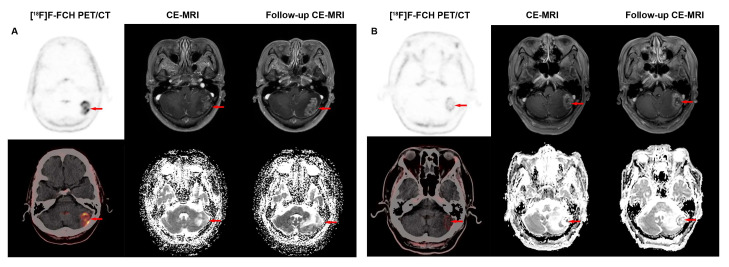
(**A**) A 64-year-old female patient with BM of lung cancer after SRS underwent follow-up examination without any symptoms. [^18^F]F-FCH PET/CT showed a lesion measuring 2.1 × 1.6 cm with high [^18^F]F-FCH uptake (SUV_max_ = 5.3) in the left cerebellar region. CE-MRI revealed that the lesion showed progressive enhancement and had a low ADC value. After 4 months of follow-up, CE-MRI revealed that the lesion showed significant enlargement and had a low ADC value. The lesion was diagnosed as recurrent neoplasms (red arrow). (**B**) A 64-year-old male patient developed headache after SRS treatment. An [^18^F]F-FCH PET/CT and MR examination revealed a left cerebellar mass. The left cerebellar mass measured 2.6 × 1.9 cm with an SUV_max_ value of 1.43. CE-MRI revealed that the lesion showed progressive enhancement and had a low ADC value. After 8 months of follow-up, CE-MRI revealed that the lesion showed no significant size changes and had a low ADC value. This lesion was finally confirmed to be a non-recurrent lesion (indicated by a red arrow).

**Figure 4:**
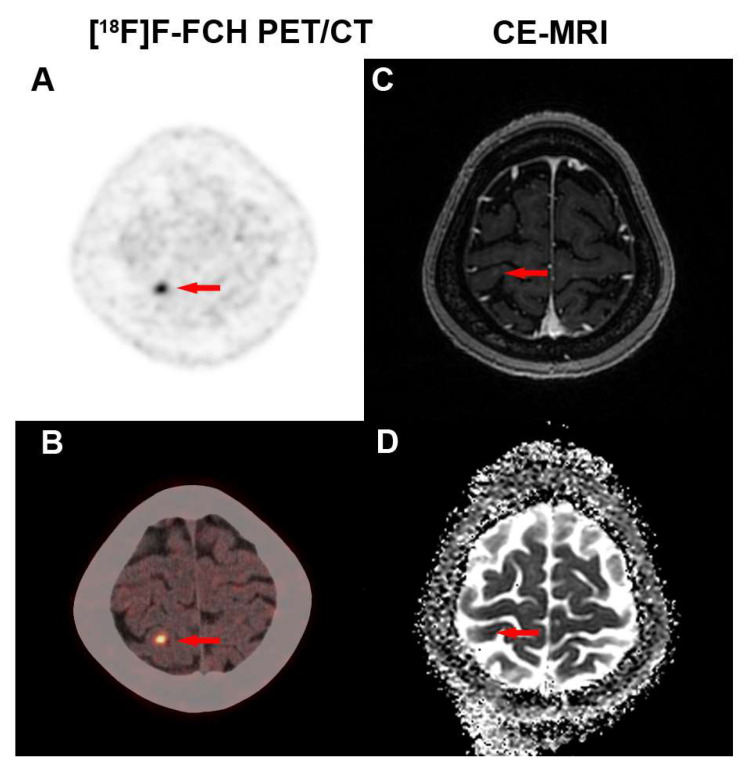
(**A**) A 52-year-old female patient with BM of lung cancer after SRS underwent follow-up examination without any symptoms. (**A**,**B**) [^18^F]F-FCH PET/CT showed a lesion with 0.4 cm in diameter and a high [^18^F]F-FCH uptake (SUV_max_ = 4.1) in the right parietal lobe [(**A**) (PET image), (**B**) (PET/CT fusion image), red arrow]. (**C**,**D**) CE-MRI found an enhancement lesion ((**C**), red arrow) without low ADC value ((**D**), red arrow) in the right parietal lobe, but did not make a definite diagnosis. The lesion was diagnosed as recurrent neoplasms.

In terms of lesion sizes, the results for the accuracy of two imaging modalities in different sizes are shown in Table 3. When the short-diameter of lesion was >1.0 cm and <2.0 cm, [^18^F]F-FCH PET/CT demonstrated significantly higher accuracy than CE-MRI in detecting recurrent lesions (93.33% vs. 46.67%, *p* = 0.016). The typical [^18^F]F-FCH PET/CT and MRI images of recurrent and non-recurrent lesions are shown in Figure 3. For lesions > 2.0 cm and <3.0 cm, as well as those <1.0 cm, [^18^F]F-FCH PET/CT showed numerically higher accuracy than CE-MRI (75.00% vs. 50.00% and 89.47% vs. 73.68%, *p* > 0.05), though the results were not statistically significant. Only when lesions exceeded 3.0 cm did CE-MRI exhibit marginally higher accuracy than [^18^F]F-FCH PET/CT, but this difference also lacked statistical significance (75.00% vs. 87.50%, *p* = 0.38). The smallest recurrent lesion detected by both [^18^F]F-FCH PET/CT and CE-MRI had a diameter of 0.4 cm; however, it was considered a recurrence due to the SUV_max_ of 4.1 on [^18^F]F-FCH PET/CT, while it was a negative lesion on CE-MRI (Figure 4).

### 3.4. Correlation Between Clinical Features, Parameters of [^18^F]F-FCH PET/CT, CE-MRI, and iPFS

**Figure 5:**
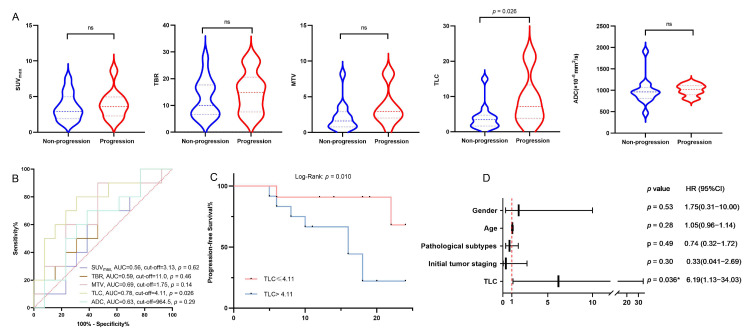
(**A**) The difference between [^18^F]F-FCH PET/CT parameters and ADC of CE-MRI in non-progression and progression groups, where only TLC values show a statistically significant difference (*p* = 0.026). No significant differences were observed in SUVmax, TBR, MTV, and ADC values. (**B**) The ROC curves of [^18^F]F-FCH PET/CT parameters and ADC of CE-MRI for progression-free survival. The optimal TLC cut-off value for distinguishing progression and non-progression groups was 4.11, with an AUC of 0.82. Sensitivity and specificity were 80% and 69%, respectively. (**C**) The Kaplan–Meier plots of iPFS for demonstrating the prognostic value of TLC analyses based on the indicated ROC curve cut-off values. (**D**) Multivariate Cox regression analysis forest plot for iPFS. After adjusting for clinical confounders, only TLC emerged as an independent predictor of iPFS. * *p* < 0.05; ns: not significant.

All thirty-one enrolled patients underwent clinical follow-up for 4–50 months (median: 20.5 months). Among these, 23 patients with metastatic lesions received post-intervention follow-up for 5–24 months (median: 16 months). Progression occurred in 13/23 patients (56.5%), whereas 10 patients (43.5%) maintained intracranial stability throughout follow-up. A statistically significant intergroup disparity was observed in TLC values (progression: 9.06 ± 2.24; non-progression: 4.01 ± 1.03; *p* = 0.026) (Figure 5A). Neither [^18^F]F-FCH PET/CT-derived metrics (SUV_max_, TBR, MTV) nor MRI-based ADC values demonstrated significant differences (Figure 5B). The optimal TLC threshold for progression prediction was 4.11 (AUC = 0.82; sensitivity and specificity were 80% and 69%, respectively). Kaplan–Meier survival analysis stratified by the TLC cut-off of 4.11 revealed a marked disparity in iPFS, with the TLC > 4.11 subgroup demonstrating inferior survival outcomes relative to the TLC ≤ 4.11 group (log-rank *p* = 0.010) (Figure 5C). As shown in Table 4, univariate analysis demonstrated that there was no significant correlation between iPFS and clinical parameters, including pathological subtypes and initial tumor staging. In the univariate analysis (Table 4), we found a significant correlation between iPFS and TLC (HR [hazard ratio] = 5.50, 95% CI [confidence interval] = 1.13–26.76, *p* = 0.035), whereas there was no significant correlation between iPFS and other parameters. In the multivariate analysis, TLC had an impact on the iPFS of patients (HR [hazard ratio] = 6.19, 95% CI [confidence interval] = 1.13–34.03, *p* = 0.036) (Table 4 and Figure 5D), suggesting that TLC was a significantly negative prognostic factor for iPFS.

## 4. Discussion

This retrospective study evaluated the clinical value of [^18^F]F-FCH PET/CT in the detection of recurrence and prognosis prediction in LCBM after SRS. By assessing 31 patients, we found that [^18^F]F-FCH PET/CT had a significantly superior performance, with high specificity and accuracy in detecting recurrence in LCBM after SRS compared to CE-MRI due to a high uptake of [^18^F]F-FCH in the recurrent lesions of BM. Regardless of [^18^F]F-FCH PET/CT or CE-MRI parameters, the recurrence groups were significantly different from those in the no-recurrence groups. However, only TLC was significantly correlated with iPFS; it was a significant prognostic factor for iPFS.

Conventional MRI with and without gadolinium contrast has currently become the standard neuro-oncological imaging modality. Although conventional MRI protocols, such as three-dimensional (3D) T1, axial fluid-attenuated inversion recovery (FLAIR), axial gadolinium contrast-enhanced T2, and 3D gadolinium contrast-enhanced T1, are useful for initial assessment, they have shown some limitations when evaluating the tumor extent, predicting the grade, and assessing the treatment response [19,20]. DWI directly measures water mobility, providing functional data on tumors and their microenvironment [37]. ADC values, derived from DWI, indicate the magnitude of diffusivity by measuring the restriction of water molecules at differing degrees of diffusion weighting [20]. Hypointense ADC values or low ADC values demonstrate decreased water diffusivity secondary to tumor cellularity, useful for differentiating the tumor type and grade [37,38]. However, ADC sensitivity is limited by edema in low-grade tumors or post-therapy scenarios [37]. Bozdağ M, et al. [39] noted significantly lower ADC entropy in SCLC brain metastases versus adenocarcinoma and squamous cell carcinoma, though ADC skewness and kurtosis showed no intergroup differences. PWI MRI demonstrates 70–100% sensitivity and 95–100% specificity in some studies [40,41,42], though these retrospective findings require histological validation in larger cohorts. In summary, there are few data on the use of ADC for detecting the recurrence of BM of lung cancer after SRS. Data on the utility of ADC for detecting lung cancer brain metastasis recurrence after stereotactic radiosurgery remain limited. This study showed that ADC in recurrence groups was significantly higher than those in non-recurrence groups, and when the cut-off value of ADC was 1002, ADC had excellent sensitivity for determining recurrent lesions. Meanwhile, CE-MRI had a 40.74% specificity and 62.96% accuracy in detecting the recurrence of BM after SRS. There were 16 false-positive lesions (59.25%) attributed to RN or scar tissue, and 4 false-negative lesions caused by tiny lesions or a partial volume effect. This may be attributed to the visual qualitative observation that both RN and recurrence exhibit diverse enhancement and restricted diffusion patterns. Only when the “centrally restricted diffusion sign” is exclusively present within the ring-enhancing lesion or combined with peripheral diffusion restriction can it significantly differentiate radiation necrosis from tumor progression [43]. When imaging voxels exceed target anatomical dimensions, particularly with small lesions, partial volume effects may prevent the detection of abnormalities [44,45]. These results indicate that ADC can indeed differentiate between recurrent and non-recurrent lesions; however, CE-MRI still exhibits limited specificity even with ADC.

Recently, [^18^F]F-FCH, a fluorinated radiotracer, has been used in the detection of brain tumors due to the low brain background and high contrast [31]. Montes A, et al. [22] reported that in 17 patients with recurrence of a primary brain neoplasm, [^18^F]F-FCH PET/CT was a true positive, suggesting that [^18^F]F-FCH PET/CT might be useful for diagnosing the recurrence of primary brain neoplasms. In this study, [^18^F]F-FCH PET/CT demonstrated a superior specificity and accuracy to CE-MRI in detecting recurrent lesions in LCBM after SRS, attributable to a significantly higher radiotracer uptake in recurrent foci. [^18^F]F-FCH PET/CT parameters, including SUV_max_, TBR, MTV, and TLC, in the recurrence groups were significantly higher than in the non-recurrence groups. However, on [^18^F]F-FCH PET/CT, there were three false-positive lesions (11.11%) with RN due to a mild uptake of [^18^F]F-FCH in the area surrounding necrosis, mimicking tumor residue or recurrence, and five false-negative lesions (18.52%) due to a partial volume effect. Although amino acid tracers demonstrate a good diagnostic performance for small tumors or RN lesions [46,47], CT-based attenuation correction (CTAC) may be influenced by CT manifestations. Cortical abnormalities can result in reduced PET signal intensity [48], necessitating future studies to address correction through advanced technical solutions or implement MR-based correction methods [49]. When the cut-off points of SUV_max_, TBR, MTV, and TLC were 2.02, 7.17, 0.73, and 1.00, respectively, [^18^F]F-FCH PET/CT had a high sensitivity and specificity in differentiating recurrent from non-recurrent lesions. However, the clinical value of these cut-off values needs to be further confirmed in future prospective studies.

The detection of tiny and occult recurrence of BM in LCBM after SRS is another challenge for the currently available imaging modalities. It is often challenging for MRI to distinguish recurrent neoplasms from treatment-related necrosis due to overlapping radiological features such as radiation necrosis, demyelination and vasogenic edema, endothelial apoptosis, and neuroinflammation [42,50]. In the current study, the accuracy of [^18^F]F-FCH PET/CT in finding recurrent lesions was significantly higher than CE-MRI for lesion sizes from 1 to 2 cm. Additionally, for lesions between 2 cm and 3 cm or <1 cm, PET shows higher specificity trends than MRI, though the results lack statistical significance. Only when the lesion exceeds 3 cm in size does MRI show a tendency toward higher specificity than PET, but this difference also does not reach statistical significance. This may be influenced by the limited sample size, but at least such a trend was indeed observed. Interestingly, the smallest recurrent lesion detected by [^18^F]F-FCH PET/CT was 0.4 cm in the margin of the frontal cortex, and it was not found by CE-MRI. These results suggest that [^18^F]F-FCH PET/CT has potential in detecting a tiny and occult recurrence of BM in LCBM after SRS compared to CE-MRI.

Regarding survival analysis, we found a significant correlation between iPFS and [^18^F]F-FCH PET parameters. The patients with higher TLC (>4.11) have a shorter iPFS than those with lower values (<4.11). García Vicente AM, et al. [51] reported that [^18^F]F-FCH PET parameters were associated with iPFS in the patients with high-grade glioma. In the univariate analysis, TLC was the only factor influencing iPFS rather than any other parameters, including ADC. In the multivariate analysis, TLC was an independent prognostic factor for iPFS. Additionally, clinical variables assessed in this research, such as gender, age, pathological subtypes, and initial tumor staging, demonstrated no significant association with iPFS. These results indicated that [^18^F]F-FCH PET/CT is useful to be able to predict the prognosis for recurrence of LCBM after SRS. Furthermore, multiple studies demonstrate the superiority of TLC over MTV and SUV values as PET-derived prognostic indicators in oncology [52]. Research across multiple cancer types has emphasized the impact of the metabolic tumor volume and radiotracer uptake on the tumor prognosis and grading [53,54,55]. This study demonstrates the novel utility of TLC in predicting intracranial recurrence and iPFS in LCBM, potentially enhancing clinical decision-making for affected patients.

This study has several limitations. Most notably, the relatively small patient population constrained the generalizability of our findings and introduced potential biases. Nevertheless, these factors do not compromise the validity of our findings for the following reasons. The experiment utilized a representative sample that largely captured the population’s characteristics. Furthermore, the analysis employed the Mann–Whitney U test, which is particularly sensitive to distributional differences, relatively robust to outliers, and especially appropriate for smaller sample sizes [56]. Additionally, the limited follow-up duration and the restricted sample size with histopathologically confirmed diagnoses may have affected the reliability of our results. Future studies will incorporate longitudinal follow-up and expanded cohort recruitment to strengthen histopathological correlation. Thirdly, as perfusion imaging is not included in the routine CE-MRI sequences, the analysis of MR perfusion scans is lacking. This aspect will be incorporated into future research through prospective trials. Finally, the prediction of intracranial progression-free status was solely evaluated based on post-SRS treatment lesions, lacking a characterization of baseline lesion features. Future studies with expanded cohorts and prospective designs are warranted to obtain more robust evidence for clinical application.

## 5. Conclusions

[^18^F]F-FCH PET/CT is a promising imaging modality for detecting the recurrence of brain metastases in lung cancer patients with brain metastasis after stereotactic radiosurgery. A high TLC is an adverse prognostic factor for intracranial progression-free survival. [^18^F]F-FCH PET/CT and CE-MRI are complementary, and their combined application has the potential to improve the diagnostic performance for the recurrence of brain metastases and the management of patient treatment. Certainly, due to the limited sample size of this study, the generalizability of the conclusions remains to be validated through future multicenter studies with large samples.

## Figures and Tables

**Table 1:** The demographic and clinical characteristics of patients.

Characteristics	Values (*n* = 31)
Sex	
M	17 (54.84%)
F	14 (45.16%)
Age (year)	54 (35–77)
<60	13 (41.94%)
≥60	18 (58.06%)
Pathological subtypes	
Non-small-cell lung cancer	25 (80.66%)
Small-cell lung cancer	6 (19.35%)
Initial tumor staging (ITS)	
I	1 (3.22%)
II	6 (19.35%)
III	13 (41.94%)
IV	11 (35.48%)
Previous systemic treatment	
Targeted therapy	5 (16.13%)
Chemotherapy	12 (38.71%)
Chemotherapy + targeted therapy	14 (45.16%)
Neural symptoms	
Yes	16 (51.61%)
Headaches	8 (25.80%)
Seizures	4 (12.90%)
Nausea and vomiting	4 (12.90%)
No	15 (48.39%)
Method of confirmation	
Pathological results	9 (29.03%)
Follow-up results	22 (70.97%)

**Table 2:** Comparison of diagnostic performance of [^18^F]F-FCH PET/CT and CE-MRI in detecting recurrence of BM in LCBM after SRS (total lesions (*n* = 54)).

Parameter (*n*)	^18^F-FCH PET/CT *n*, %, [95% CI]	CE-MRI *n*, %, [95% CI]	*p*-Value
Sensitivity (*n* = 27)	24 (88.89%) [76.22~101.56]	23 (85.19%) [70.86~99.51]	0.338
Specificity (*n* = 27)	22 (81.48%) [65.82~97.14]	11 (40.74%) [20.93~60.55]	0.003 **
Accuracy (*n* = 54)	46 (85.19%) [75.40~94.97]	34 (62.96%) [49.66~76.27]	0.008 **

** *p* < 0.001.

**Table 3:** Comparison of detective accuracy of [^18^F]F-FCH PET/CT and CE-MRI in detecting recurrent lesions of BM in LCBM after SRS based on sizes of lesions.

Short-Diameter Lesions (cm)	No. of Lesions	[^18^F]F-FCH PET/CT	CE-MRI	*p*-Value
		Accurate Lesions	Accurate Lesions	
Total	54	46 (85.19%)	34 (62.96%)	0.008 *
Φ ≥ 3	8	6 (75.00%)	7 (87.50%)	0.38
2 ≤ Φ <3	12	9 (75.00%)	6 (50.00%)	0.38
1 ≤ Φ <2	15	14 (93.33%)	7 (46.67%)	0.016 *
Φ < 1	19	17 (89.47%)	14 (73.68%)	0.42

* *p* < 0.05.

**Table 4:** Univariate and multivariate analysis of clinical characteristics, [^18^F]F-FCH PET/CT parameters, and ADC of CE-MRI for iPFS.

Variable	Univariate Analysis			Multivariate Analysis		
	HR	95% CI	*p*-Value	HR	95% CI	*p*-Value
Clinical parameters						
Gender	0.65	0.74–0.21	0.64	1.75	0.31–10.00	0.53
Age	1.03	0.97–1.09	0.38	1.05	0.96–1.14	0.28
Pathological subtypes	1.04	0.48–2.24	0.93	0.74	0.32–1.72	0.49
Initial tumor staging	2.89	0.36–22.78	0.32	0.33	0.04–2.69	0.30
[^18^F]F-FCH PET/CT parameter						
TLC	5.50	1.13–26.76	0.035 *	6.19	1.13–34.03	0.036 *
CE-MRI parameter						
ADC	0.27	0.55–8.48	2.16	/	/	/

* *p* < 0.05.

## Data Availability

The datasets used and/or analyzed during the current study are available from the corresponding author on reasonable request.

## Abbreviations

The following abbreviations are used in this manuscript:

PET/CT	Positron emission tomography/computed tomography
FCH	Fluorocholine
CE	Contrast-enhanced
MRI	Magnetic resonance imaging
iPFS	Intracranial progression-free survival
BM	Brain metastasis
SRS	Stereotactic radiosurgery
BBB	Blood–brain barrier
RN	Radiation necrosis
LCBM	Lung cancer brain metastasis
SUV_max_	Standardized uptake value
MTV	Metabolic tumor volume
TLC	Total lesion of choline uptake
ADC	Apparent diffusion coefficient
DWI	Diffusion-weighted imaging
ROC	Receiver operating characteristic
ROI	Region of interest
AUC	Area under the ROC curve
